# The Temperature Mode of the Carbon-Carbon Multi-Disc Brake in the View of the Interrelations of Its Operating Characteristics

**DOI:** 10.3390/ma13081878

**Published:** 2020-04-16

**Authors:** Aleksander A. Yevtushenko, Piotr Grzes, Adam Adamowicz

**Affiliations:** Faculty of Mechanical Engineering, Bialystok University of Technology (BUT), 15-351 Bialystok, Poland; a.yevtushenko@pb.edu.pl (A.A.Y.); a.adamowicz@pb.edu.pl (A.A.)

**Keywords:** braking, frictional heating, temperature, multi-disc brake, carbon, carbon frictional composite material

## Abstract

In this paper, a methodology for conducting a computer simulation of the frictional heating process of a multi-disc braking system is proposed. The single braking of a system of three identical discs made of carbon–carbon (C/C) carbon frictional composite material (CFCM) is considered. In order to determine the operational characteristics of the brake, a heat dynamics of friction (HDF) system of equations is formulated, which takes into account the contact pressure rise time, thermal sensitivity of the C/C material, the change in the coefficient of friction during braking, the parameters of the friction surface’s microgeometry and the mutual influence of sliding velocity and temperature. A numerical solution using the finite element method (FEM) of the HDF system of equations allows us to determine changes in key braking process characteristics, such as work done, braking torque, friction coefficient, heat transfer coefficient, velocity and temperature. Finally, a comparative analysis of the results obtained for three different time profiles of the coefficient of friction is carried out.

## 1. Introduction

Single-name friction pairs made of carbon–carbon (C/C) carbon frictional composite material (CFCM) are widely used in highly loaded friction systems—most often in the multi-disc brakes of civil and military aircrafts [1,2]. Due to their high specific heat capacity, low density, good friction properties and wear resistance, CFCMs are used in heavy temperature braking modes when volume and surface temperatures exceed 800 and 1500 °C, respectively [3,4].

Even compared with ceramic metal frictional materials (CMFM), CFC materials subjected to high clamping forces and high speeds of friction sliding do not lead to significant increases in the wear of the mating surfaces [5,6]. One of the important advantages of CFCM is the lack of the phenomenon of brake disc tacking, which often occurs in CMFM. Depending on the purpose, as well as the material price, the thermophysical, mechanical and frictional properties may differ.

Experimental studies, including full-size bench tests of braking systems with CFCM discs, have shown that, as with CMFM, the main parameter in assessing the wear resistance of the disc material, and thus the brake’s durability (lifespan), is the temperature mode [7,8,9]. Therefore, the development of effective and reliable mathematical models to find transient temperature distributions in multi-disc brakes is a current scientific problem.

It should be noted that CFCMs are characterized by the coefficient of friction’s significant dependence on temperature. In the case of the Termar type of CFCM, the coefficient of friction during cold braking is equal to 0.1 and when heated to 200 °C ÷ 300 °C, it reaches 0.5 ÷ 0.7, and maintains this level up to a critical temperature of about 1000 °C [10]. Therefore, the frictional properties of the pair made of CFCM will significantly depend on the temperature mode of the braking process. It is known that the maximum temperature has a decisive influence on the coefficient of friction, which is the sum of the mean temperature of the nominal contact area (region) of the discs and flash temperature—the temperature of the real contact area [11,12].

The temperature dependence of the coefficient of friction when determining the temperature mode of a multi-disc brake can be taken into account by means of the heat dynamics of friction (HDF) system of equations [13,14,15]. The system of equations of HDF for the single braking consists of the following basic components (blocks):

(1) The law for changing the contact pressure during braking;
(2) Formulas approximating the experimental data, concerning the temperature dependence of the coefficient of friction, thermal conductivity, specific heat capacity, as well as the hardness of friction pair materials;
(3) The initial value problem for the equation of motion of a multi-disc system at a given value of the work done during braking;
(4) The boundary value heat conduction problem, taking into account heat generation as a result of friction in the nominal contact area of two adjacent discs (the thermal problem of friction);
(5) The calculation of the flash temperature based on the experimental formulas;
(6) The temperature summation hypotheses.

It should be noted that the statements of each issue from points (3–5) include contact pressure and the coefficient of friction. If the evolution of the contact pressure during braking is determined a priori, then the coefficient of friction can be constant (averaged during braking) or varied depending on the temperature. In the first case, the scheme for determining changes in the key parameters during braking, such as velocity and temperature, based on the solution of the above-mentioned system of equations of HDF, involves the sequential implementation of the following steps:

(1) Determining the friction force at the given constant values of the coefficient of friction and the nominal field of the contact area, as well as the contact pressure changing over time with subsequent substitution to the right-hand side of the equation of motion;
(2) Solving the initial value problem for the equation of motion with a known friction force time profile in order to determine the change in velocity and braking time;
(3) Determining the temporal profile of the friction power density—the product of the constant coefficient of friction, the change in contact pressure and sliding velocity during braking;
(4) Determining the evolution of the mean temperature of the nominal contact area at a known time profile of the friction power density, flash temperature and maximum temperature of the braking system.

This approach, to determine the maximum temperature of the system, consisting of two extreme stationary (stators) and one central rotating (rotor) disc made of CFCM Termar-ADF, was implemented in [16,17,18]. The mean temperature of the nominal contact area was determined on the basis of an exact analytical solution to a one-dimensional thermal problem of friction [16,17] or a numerical solution, using FEM, to solve an axisymmetric (2D) problem [18].

Nevertheless, the abovementioned scheme for determining the temperature mode of a multi-disc brake has a significant disadvantage—it does not take into account the mutual influence of sliding velocity and temperature, and the use of a constant coefficient of friction value that is averaged during braking, which can lead to significant discrepancies between the corresponding theoretical and experimental temperature values. This is particularly noticeable in severe friction modes when a significant amount of heat is generated, as changes in the coefficient of friction during braking can result in an increase of 200% ÷ 400% from its initial value [19,20]. The basic elements, taking into account the mutual relations of the above parameters, are the experimental data regarding the dependence of the coefficient of friction on temperature. One way of obtaining the experimental data of the dependence of the coefficient of friction on temperature for the single-name CFCM pairs is testing for frictional thermal stability in conditions of stationary friction [21]. In such tests, the mean temperature of the nominal contact area is controlled using thermocouples installed near that area, the flash temperature is calculated theoretically, and the maximum temperature is determined using a special methodology [22]. This methodology is based on the X-ray measurement of the CFCM crystal structure parameter, which is the distance between planes before and after friction tests [23]. As a result, for the single-name CFCM friction pairs, the specified dependencies of the coefficient of friction on the maximum temperature at a given contact pressure are obtained. The disadvantage of using the curves obtained in this way, when modeling the brake temperature field using the HDF system of equations is that when the value of the nominal pressure changes, the experimental tests must be re-performed using the abovementioned method. Therefore, a test procedure was developed for testing the single-name CFCM pairs for thermal impulse (non-stationary) one- or two-sided friction using typical ring samples and taking into account the change in velocity from the initial maximum value to zero at the time of stopping [24]. The test procedure included three model braking modes (500 cycles of braking in each mode) at different nominal pressure values achieved at the same rise time and constant values of initial velocity and total friction (braking) work. Such tests allowed us to obtain the three-parameter functions of the dependence of the coefficient of friction on the nominal pressure, sliding velocity and maximum temperature.

The 3D simulation tests were carried out to determine the temperature mode of the pad–disc braking system covering six different brake pad materials (three represent asbestos friction materials—two belong to the CMFM class and one is Retinax type A—a composite based on phenol–formaldehyde resins) related sequentially with a cast-iron disc [25]. These calculations concerned a light brake temperature mode when the friction surface temperature did not exceed 200 °C. The continuation of that research is realized in the results presented in this study of the heavy mode of the multi-disc brake temperature (with the maximum temperature reaching 750 °C and higher), consisting of three identical discs made of CFCM-5. Based on the axisymmetric (2D) numerical solution, the HDF system of equations was studied, taking into account the mutual effect of changing the main characteristics of the braking process, such as coefficient of friction, work done, torque, braking time, sliding velocity, convection cooling and maximum temperature. A comparative analysis of these characteristics, taking into account three variants of the coefficient of friction changing in the braking process, was carried out.

## 2. System of Equations of Heat Dynamics of Friction (HDF)

**Figure 1:**
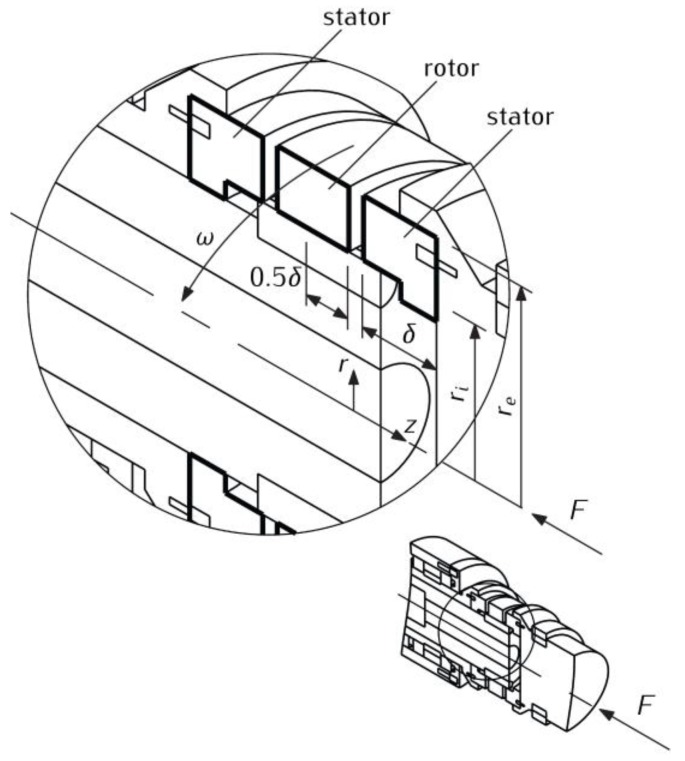
Schematic diagram of the friction pair on the test rig.

*Contact pressure change during braking.* Consider the friction pair, consisting of three identical ring discs, located concentrically along the axis of rotation, according to Figure 1. The central disc rotates at a constant angular velocity $\omega_{0}$, and the outer discs are stationary. At the initial time moment *t* = 0, the outer discs are pressed against the middle disc with a linear increase in contact pressure *p* from zero to the value *p*_0_:

(1) $$p(t)=p_{0}p^{*}(t),\;p^{*}(t)=(t/t_{i})H(t_{i}-t)+H(t-t_{i}),\;0\leq t\leq t_{s},$$

where *t_i_* is the rise time of the contact pressure, *t_s_* is the braking time and H(⋅) is the Heaviside step function.

*Approximating functions*. The applied mechanical load, which resists the motion of the rotating disc due to the friction force, leads to the conversion of kinetic energy into thermal energy, causing the entire system to heat up. We assume that the discs are made of the same friction carbon composite, whose effective properties change under the influence of temperature *T.* Experimental data on such changes for the thermal conductivity *K*, specific heat capacity *c* and Brinell hardness *HB* are approximated by the functions:

(2) $$K(T)=K_{0}K^{*}(T),\;c(T)=c_{0}c^{*}(T),\;HB(T)=HB_{0}HB^{*}(T),$$

(3) $$K_{0}\equiv K(T_{0}),\;c_{0}\equiv c(T_{0}),\;HB_{0}\equiv HB(T_{0}),$$

(4) $$K^{*}(T)=K_{1}+\frac{K_{2}}{{\left[K_{3}(T-T_{K_{1}})\right]}^{2}+1}+\frac{K_{4}}{{\left[K_{5}(T-T_{K_{2}})\right]}^{2}+1},$$

(5) $$c^{*}(T)=c_{1}+\frac{c_{2}}{{\left[c_{3}(T-T_{c_{1}})\right]}^{2}+1}+\frac{c_{4}}{{\left[c_{5}(T-T_{c_{2}})\right]}^{2}+1},$$

(6) $$HB^{*}(T)=HB_{1}+\frac{HB_{2}}{{\left[HB_{3}(T-T_{HB_{1}})\right]}^{2}+1}+\frac{HB_{4}}{{\left[HB_{5}(T-T_{HB_{2}})\right]}^{2}+1},$$

where *T*_0_ is the initial system temperature and *K_i_*, *c_i_*, *HB_i_*, *i* = 1,2,3,4,5, *T_Ki_*, *T_ci_*, *T_HBi_* and *i* = 1,2 are the constants, whose selection is based on the specific methodologies [26]. The density $\rho_{0}$ of the discs’ material remains unchanged.

*Initial value problem for the equation of motion.* In addition to frictional heating, an increase in pressure initiates the braking process—a reduction in the angular velocity *w* of the central disc from the initial value *w*_0_ to zero at the time of stopping *t* = *t_s_*. The change in angular velocity in the braking process can be found from the solution of the following initial value problem for the equation of motion [17]:

(7) $$I_{0}\frac{d\omega (t)}{dt}=-2M(t),\;0\leq t\leq t_{s},\;\omega (0)=\omega_{0},$$

where

(8) $$M(t)=F(t)r_{eq},\;F(t)=f(t)p(t)A_{a},\;A_{a}=\pi (r_{e}^{2}-r_{i}^{2}),$$

(9) $$I_{0}=\frac{2W_{0}}{\omega_{0}^{2}},\;r_{eq}=\frac{2(r_{e}^{2}+r_{i}r_{e}+r_{i}^{2})}{3(r_{e}+r_{i})},$$

where *f* is the friction coefficient, *W*_0_ is the initial kinetic energy of the rotating masses and *r_i_* and *r_e_* are the internal and external radii of the discs, respectively. Equations (7)–(9) present the following solution:

(10) $$\omega (t)=\omega_{0}-2I_{0}^{-1}\int_{0}^{t}M(\tau )d\tau ,\;0\leq t\leq t_{s},$$

where the braking time *t_s_* is determined by checking the condition at the end of the process:

(11) $$\omega (t_{s})=0.$$

*Thermal problem of friction.* In the contact area, the conditions of perfect thermal friction contact were adopted, i.e., the temperature of the discs on the friction surfaces are equal, and the sum of the density of heat fluxes directed perpendicularly from the contact surface inside each disc is equal to the specific friction power [27]. The convection mode of heat transfer takes place on the free surfaces of discs, with the surrounding environment at a temperature *T_a_* and with a variable heat transfer coefficient *h* [28,29]:

(12) $$h(t)=C\;V_{eq}^{0.8}(t),\;V_{eq}(t)=\omega (t)r_{eq},\;0\leq t\leq t_{s},\;C=10.318\;\mathrm{kg}\;m^{-0.8}\;s^{-2.2}{\;K}^{-1}.$$

**Figure 2:**
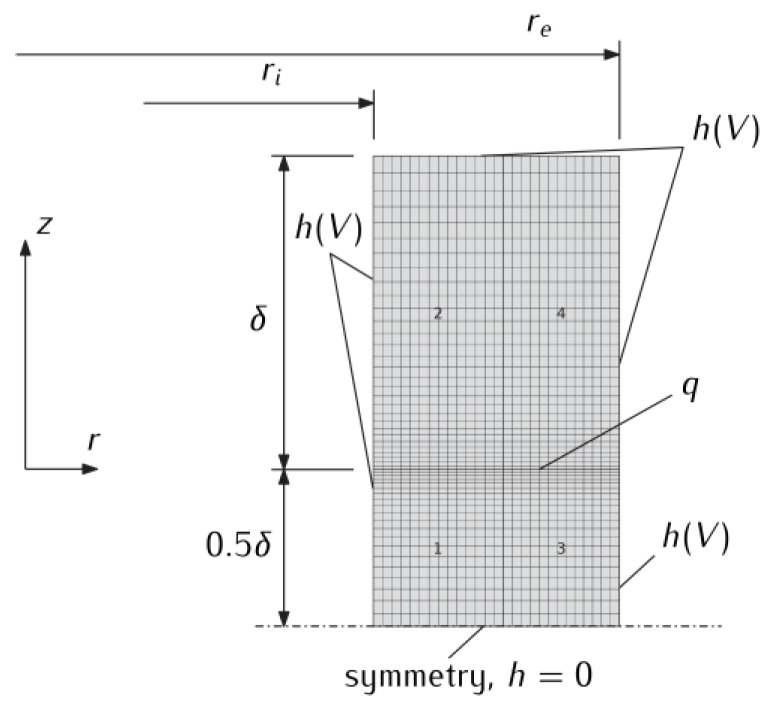
Finite element mesh of the three-element braking system with boundary conditions.

Due to the geometrical and force symmetry, to determine the temperature in a system with three identical discs, it is enough to consider a system consisting of one stationary disc with a thickness δ and a movable disc with a thickness 0.5δ (Figure 2). Then, referring such a system to cylindrical coordinates *r*, *z*, the transient temperature field *T*(*r*, *z*, *t*) can be found from the solution of the following axisymmetric boundary value problem of heat conduction:

(13) $$\frac{1}{r}\frac{\partial }{\partial r}\left[rK(T)\frac{\partial T}{\partial r}\right]+\frac{\partial }{\partial z}\left[K(T)\frac{\partial T}{\partial z}\right]=\rho_{0}c(T)\frac{\partial T}{\partial t},\;r_{i}<r<r_{e},-0.5\delta <z<\delta ,0<t\leq t_{s},$$

(14) $$T(r,0^{+},t)=T(r,0^{-},t),\;r_{i}<r<r_{e},\;0<t\leq t_{s},$$

(15) $${K(T)\frac{\partial T}{\partial z}|}_{z=0^{-}}-{K(T)\frac{\partial T}{\partial z}|}_{z=0^{+}}=q(r,t),\;\;r_{i}<r<r_{e},\;0<t\leq t_{s},$$

(16) $$K{(T)\frac{\partial T}{\partial z}|}_{z=\delta }=h(t)[T_{a}-T(r,\delta ,t)],\;\;r_{i}<r<r_{e},\;0<t\leq t_{s},$$

(17) $$K{(T)\frac{\partial T}{\partial r}|}_{r=r_{i}}=h(t)[T(r_{i},z,t)-T_{a}],\;0<z<\delta ,\;0<t\leq t_{s},$$

(18) $$K{(T)\frac{\partial T}{\partial r}|}_{r=r_{e}}=h(t)[T_{a}-T(r_{e},z,t)],\;0<z<\delta ,\;0<t\leq t_{s},$$

(19) $${\frac{\partial T}{\partial z}|}_{z=-0.5\delta }=0,\;r_{i}<r<r_{e},\;0<t\leq t_{s},$$

(20) $$K{(T)\frac{\partial T}{\partial r}|}_{r=r_{i}}=h(t)[T(r_{i},z,t)-T_{a}],-0.5\delta <z<0,\;0<t\leq t_{s},$$

(21) $$K{(T)\frac{\partial T}{\partial r}|}_{r=r_{e}}=h(t)[T_{a}-T(r_{e},z,t)],-0.5\delta <z<0,\;0<t\leq t_{s},$$

(22) $$T(r,z,0)=T_{0},\;\;\;r_{i}\leq r\leq r_{e},\;\;\;-0.5\delta \leq z\leq \delta ,$$

where the friction power density in the boundary condition (Equation (15)) is:

(23) $$q(r,t)=f(t)p(t)r\omega (t),\;\;r_{i}<r<r_{e},\;0<t\leq t_{s}.$$

The time profile of the temperature, averaged in the contact area, can be determined from the formula:

(24) $$T_{m}(t)=A_{a}^{-1}\int_{r_{i}}^{r_{e}}T(r,0,t)rdr,\;0\leq t\leq t_{s}.$$

*Flash temperature T_f_* occurs in real contact areas and can be regarded as additional value to the mean temperature *T_m_* (Equation (24)). In the case of the plastic mechanism of roughness deformation, the evolution of flash temperature during braking was determined by the following equation [11,12]:

(25) $$T_{f}(t)=\frac{1.73q(r_{eq},t)d_{r}(t)A_{a}}{\left[4K(t)+\sqrt{\pi \rho_{0}V_{eq}(t)\;d_{r}(t)K(t)c(t)}\right]A_{r}(t)},\;0\leq t\leq t_{s},$$

where changes in the diameter *d_r_* of the average statistical real contact area and the total field of such areas *A_r_* describe the relationships [30,31]:

(26) $$d_{r}(t)=2{\left(\frac{2r_{av}h_{\max}}{v}\right)}^{\frac{1}{2}}{\left[\frac{p(t)}{HB(t)b_{0}}\right]}^{\frac{1}{2\nu }},\;A_{r}(t)=\frac{p(t)A_{a}}{HB(t)},$$

(27) $$K(t)\equiv K[T_{m}(t)],\;c(t)\equiv c[T_{m}(t)],\;HB(t)\equiv HB[T_{m}(t)],$$

where *r_av_* and *h_max_* are the mean rounding radius of the roughness vertices and their maximum height, respectively, *v* and *b*_0_ are the parameters of the reference surface curve obtained from profilograms in the longitudinal and transverse direction of the friction surfaces of the discs, according to the specific methodology [32].

*Temperature summation hypothesis.* The change in the maximum temperature of the considered system in the braking process was determined from the following equation [13,14,15]:

(28) $$T_{\max}(t)=T_{m}(t)+T_{f}(t),\;0\leq t\leq t_{s},$$

where *T_m_* is the mean temperature of the nominal contact area (Equation (24)), and *T_f_* is the flash temperature (Equations (25)–(27)).

## 3. Solution of HDF System of Equations

The numerical solution of the HDF system of equations formulated above was obtained using the finite elements method. For this purpose, COMSOL Multiphysics^®^ software (v.5.3, www.comsol.com, COMSOL AB, Stockholm, Sweden) was used, enabling the calculation of mutually coupled physical processes. The finite element mesh of the analyzed three-disc braking system with the adopted boundary conditions is shown in Figure 2. The number of quadratic Lagrange quadrilateral elements is equal to 1350, while the number of degrees of freedom in the model is 5555.

The calculation procedure consisted of the execution of the following steps:

(1) Defining the contact pressure profile in the contact area of the discs (Equation (1));
(2) Determining the coefficients in the formulas approximating the dependence of thermophysical properties and hardness of materials on the temperature (Equations (2)–(6)) for the selected disc material;
(3) For a fixed time step in the braking process, simultaneously solving the following issues:
(a) The initial value problem for the equation of motion (Equations (7)–(11));
(b) The thermal problem of friction (Equations (13)–(24));
(c) The determination of the flash temperature (Equations (25)–(27)).
(4) Finding the most important frictional characteristics of the braking system in question—braking time, change in velocity during braking, maximum temperature, friction torque, friction work and heat transfer coefficient.

It should be noted that the velocity depends on the coefficient of friction, and to determine the temperature it is necessary to know the density of friction power in the boundary condition (Equation (15)). As follows, from Equation (23), the density of friction power also depends on the coefficient of friction. However, in the formula for calculating the flash temperature *T_f_*, there is both the a priori unknown velocity and the density of friction power. Therefore, the coefficient of friction is a key parameter that connects issues via the maximum temperature. Such relationships form a loop, which makes it impossible to solve all the problems in the same time step. This problem was solved by introducing the value of the friction coefficient from the previous time step into the formula for the flash temperature (Equation (25)). At first, however, it was solved by the value of the friction coefficient corresponding to the initial temperature.

To define the abovementioned relations, the Mathematics module was used, containing tools such as ordinary differential system of equations (ODEs) and differential-algebraic system of equations (DAEs), Auxiliary Contributions (e.g., Discretization, Weak Constraint, Weak Contribution on Mesh Boundaries), Deformed Geometry, Moving Mesh Features and others. When determining the value of the friction coefficient from the previous time step, Domain ODEs and DAEs (dode) tools available in COMSOL were used. Additionally, it was necessary to include the Previous Solution in the Study group.

Furthermore, Global ODEs and DAEs were used to solve the initial value problem for the equation of Motion (Equations (7)–(10)) and Events, enabling the calculation to be completed when condition (Equation (11)) was met.

To perform numerical calculations, the Time-Dependent Solver—the Implicit Backward Differentiation Formula (BDF) method solver of the linear multistep methods, with an order of accuracy varying from one (backward Euler method) to five—was used. In order to obtain the stability of the solution, a higher order is automatically selected, then, if necessary, a lower order is taken [33].

The above method, like the others available in the program (the implicit Generalized Alpha and the explicit Runge–Kutta), allows the selection of a Free, Intermediate, Strict or Manual time step. The default option is Free, which adjusts the step to the specified tolerance. The disadvantage of this approach is the lack of control over the time points of the solution obtained and, thus, in extreme cases, sudden changes in the boundary conditions (friction power) in the heating area can be ignored. Therefore, in this simulation of friction heating, a strict time step of 0.01 s was used. This forces the determination of temperature and other changing parameters at given time points.

## 4. Numerical Analysis

**Figure 3:**
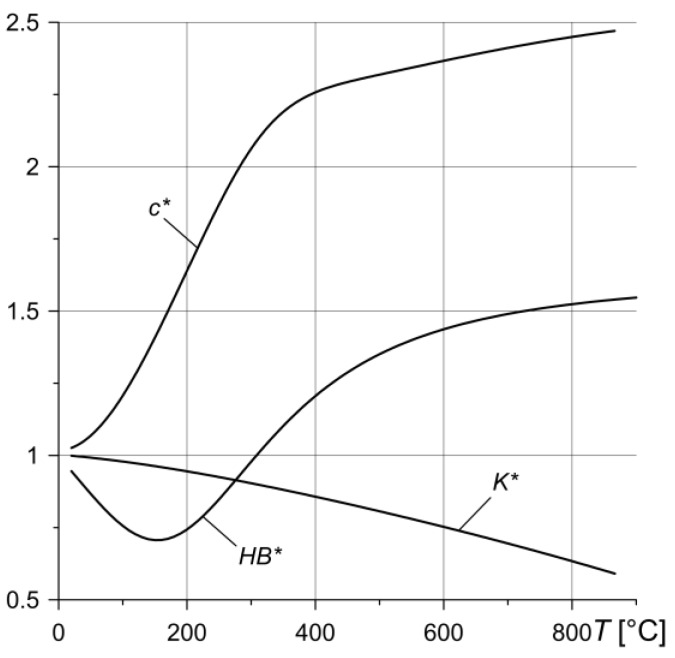
Changes in dimensionless functions K*(T), c*(T) and HB*(T) for carbon frictional composite material (CFCM)-5.

A system of three discs made of CFCM-5 was considered. The thermophysical properties at *T*_0_ = *T_a_* = 20 °C of such a material (*K*_0_ = 16.955 W·m^−1^K^−1^, *c*_0_ = 692.6 J·kg^−1^K^−1^, *HB*_0_ = 90.982 MPa, $\rho_{0}$ = 1750 kg·m^−3^) the dimensions of the discs (*r_i_* = 26.5 mm, *r_e_* = 37.5 mm, δ = 14 mm) and the values of the parameters of friction surface microgeometry (*v* = 1.5, *b*_0_ = 2.5, *r_av_* = 630 mm, *h_max_* = 2.2 μm) were adapted from [16,17,24]. The graphs of dimensionless functions K*(T), c*(T) and HB*(T) (Equations (4)–(6)) with coefficients *K_i_* and *c_i_*, listed in Table 1, are presented in Figure 3.

The calculations were made at the following input operating parameters: *t_i_* = 0.5 s, *p*_0_ = 0.45 MPa, *w*_0_ = 542.503 rad·s^−1^, *W*_0_ = 103.54 kJ [16,18]. The values of the other parameters required to perform the simulations were determined from Equations (8), (9) and (12): *r_eq_* = 32.315 mm, *A_a_* = =22.117 cm^2^, *I*_0_ = 0.7036 kg·m^2^, *V_eq_* = 17.531 m·s^−1^.

The following three calculation variants related to the variability of the coefficient of friction *f* during braking 0 < *t* ≤ *t_s_* were analyzed:

- Variant 1:
  (29) $$\begin{array}{ll} f(t)= & f[p(t),\;V_{eq}(t),\;T_{\max}(t)]=\\ & =0.163-3.9589\;p(t)-6.5837\cdot 10^{-2}V_{eq}(t)+2.564\cdot 10^{-3}T_{\max}(t)+\\ & +2.9729\;{\left[p(t)\right]}^{2}+3.936\cdot 10^{-3}{\left[V_{eq}(t)\right]}^{2}-1.353\cdot 10^{-6}{\left[T_{\max}(t)\right]}^{2}+\\ & +0.1434p(t)V_{eq}(t)-5.1849\cdot 10^{-5}V_{eq}(t)T_{\max}(t)+7.3887\cdot 10^{-4}p(t)T_{\max}(t), \end{array}$$
- Variant 2:
  (30) $$f(t)=f[T_{\max}(t)]=1.561-\frac{1.3991}{{\left\{7.077\times 10^{-4}[T_{\max}(t)-132.02]\right\}}^{2}+1},$$
- Variant 3:
  (31) $$f(t)=f_{0}=0.267,\;0<t\leq t_{s}.$$

**Figure 4:**
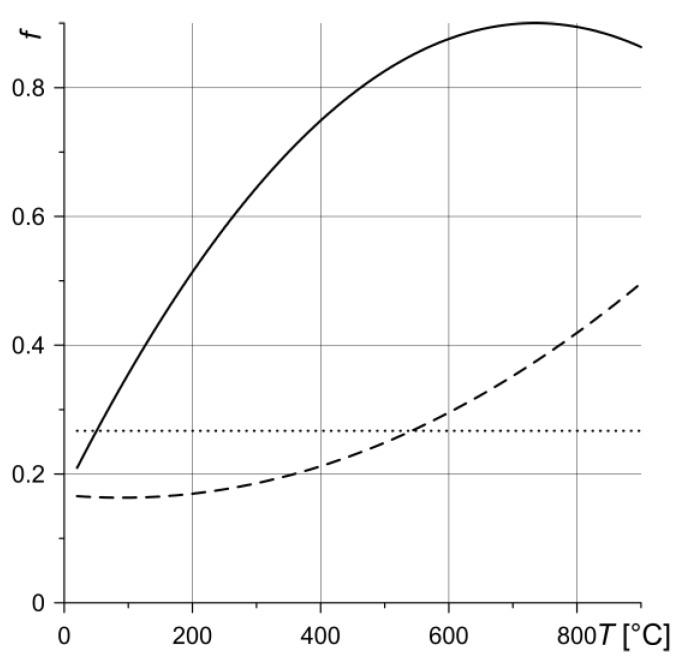
Dependence of the coefficient of friction of the single-name friction pair CFCM-5 on the maximum temperature *T_max_*: solid line—variant 1, dashed line—variant 2, dotted line—variant 3.

In the first variant, the three-parameter function of the dependence of the coefficient of friction (Equation (29)) of the CFCM-5 (the single-name friction pair) on contact pressure *p* (Equation (1)), sliding velocity *V_eq_* (Equation (12)) and maximum temperature *T_max_* (Equation (28)) was obtained on the basis of experimental tests on thermal pulse friction with model braking modes [24]. Equation (29) has been restricted to the following ranges of parameter changes: *p* = 0.196 ÷ 0.5 MPa, *V_eq_* = 0 ÷ 17.531 m·s^−1^, *T_max_* = 20 ÷ 900 °C. This article also presents a graph of the dependence of the coefficient of friction on the maximum temperature *T_max_*, obtained on the basis of experimental tests on the friction stability of that pair at nominal contact pressure *p*_0_ = 045 MPa. That plot was the basis for obtaining the approximation Equation (30) in the second variant. The value of 0.267 for the coefficient of friction in the third variant is the value of this coefficient, averaged during braking from the second variant. Diagrams of the dependence of the coefficient of friction on the maximum temperature for the three above-mentioned variants are shown in Figure 4. Here and further on, the results corresponding to the calculations according to the first variant are marked with solid lines, the second variant with dashed lines, and the third variant with dotted lines.

**Figure 5:**
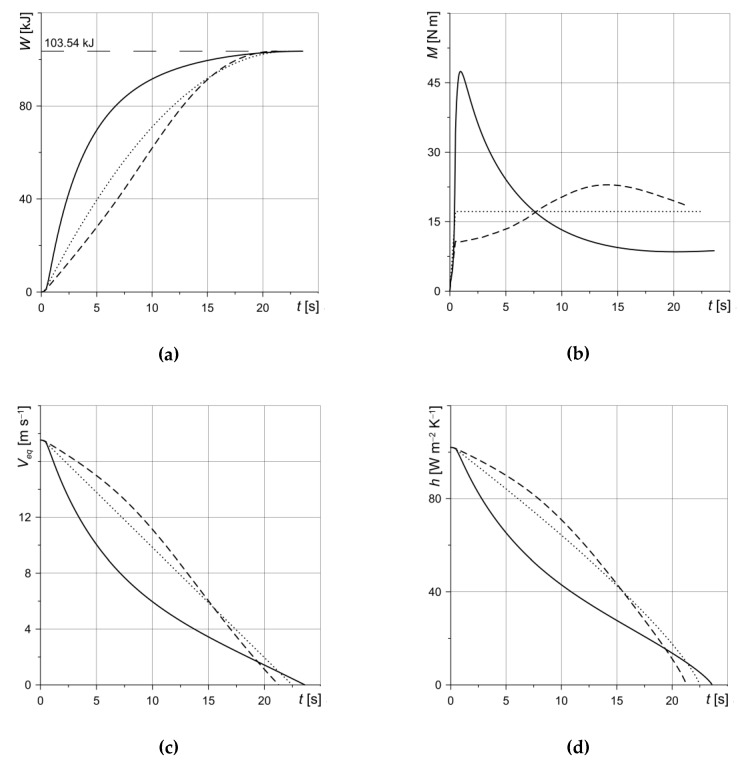
Change in: (**a**) work done *W*; (**b**) braking torque *M*; (**c**) linear velocity *V_eq_*; (**d**) heat transfer coefficient *h*(*V_eq_)* during braking. Solid line—variant 1, dashed line—variant 2, dotted line—variant 3.

Time profiles, determined as a result of numerical simulations of such important characteristics of the braking process as work done, braking torque, sliding velocity and heat transfer coefficient are presented in Figure 5.

The change in friction work (*W*) done during braking was calculated based on the following equation:

(32) $$W(t)=2A_{a}\int_{0}^{t}q(r_{eq},\tau )d\tau ,\;0\leq t\leq t_{s},$$

where *q*(*r_eq_*, *t*) is the friction power density (Equation (23)) on the equivalent radius (Equation (9)). With the passage of time, the friction work increases monotonically, reaching, at the moment of stopping, one and the same value for each calculation variant, equal to the initial rotational kinetic energy of the system *W*_0_ = 103.54 kJ (Figure 5a). An equal value for the total friction work in each of the considered calculation variants is necessary to carry out a comparative analysis of the results obtained.

The braking torque time courses *M* (Equation (8)) are shown in Figure 5b. Due to taking into account the rise time *t_i_* of the contact pressure *p* (Equation (1)) in the initial braking stage until the braking time *t = t_i_* = 0.5 s, the braking torque increases from zero to a specific value, which is the highest for the first variant and the lowest for the second variant. The braking torque time profiles over a period of time *t_i_* ≤ *t* ≤ *t_s_*, obtained when performing calculations according to three variants, are different. In the first variant, the braking torque decreases from the maximum value 47 Nm reached at the moment *t* = 1 s to the minimum value 10 N·m, *t* = 17 s, remaining practically at this value until it stops at *t_s_* = 23.6 s. When calculating the coefficient of friction with the use of a single-parameter relationship (second variant), the braking torque increases until *t* = 13 s, when it reaches its maximum value, 20 N·m. Then, a slight reduction in the braking torque to the value 13 N·m at the moment of stopping *t_s_* = 22.39 s takes place. At the constant coefficient of friction (third variant), the braking torque, after reaching the nominal value 16 N·m at *t = t_i_* = 0.5 s, remains constant until it stops *t_s_* = 22.48 s. Comparing the results presented in Figure 5b, it can be stated that the use of a three-parameter function for the coefficient of friction change over time (option one) allows us to assess the possibility of the appearance of undesirable self-oscillations in the CFCM disc set at the initial stage of the braking process. This may indicate the possibility of the occurrence of friction vibrations in the whole set of discs at that time. This is naturally an undesirable phenomenon, since such fluctuations in the resonant mode with the wheeled system and landing gear during the braking of the aircraft can lead to emergency situations upon landing [5,6]. In order to assess the frequency and amplitude of friction vibrations, the system of equations of HDF should be supplemented with the corresponding dynamics problem.

The changes in the linear velocity *V_eq_* and heat transfer coefficient *h* during braking are presented in Figure 5c,d, respectively. In the initial braking period, the velocity decreases the fastest in the calculations conducted according to the first variant, and the slowest in accordance with the second variant. However, in the final phase, we see the opposite picture—the longest braking (*t_s_* = 23.6 s) for the first variant, and the shortest (*t_s_* = 22.39 s) for the second variant. With the third option, an intermediate result was obtained *t_s_* = 22.48 s. The nature of the change in time of the linear velocity of the rotating disc *V_eq_* (Figure 5c) as a result of the relationship (Equation (12)) translates into the evolution of the heat transfer coefficient *h* (Figure 5d). The considered braking system, consisting of three identical discs, was loaded with a mass distribution, giving a moment of inertia equal to *I*_0_, and accelerated to the initial angular velocity *w*_0_ = 542.503 rad·s^−1^. The value of the heat transfer coefficient corresponding to that velocity, according to Equation (12), is equal to 102 W·m^−2^K^−1^ and is close to the limit value (100 W·m^−2^K^−1^) for the convective cooling of disc braking systems [34,35].

**Figure 6:**
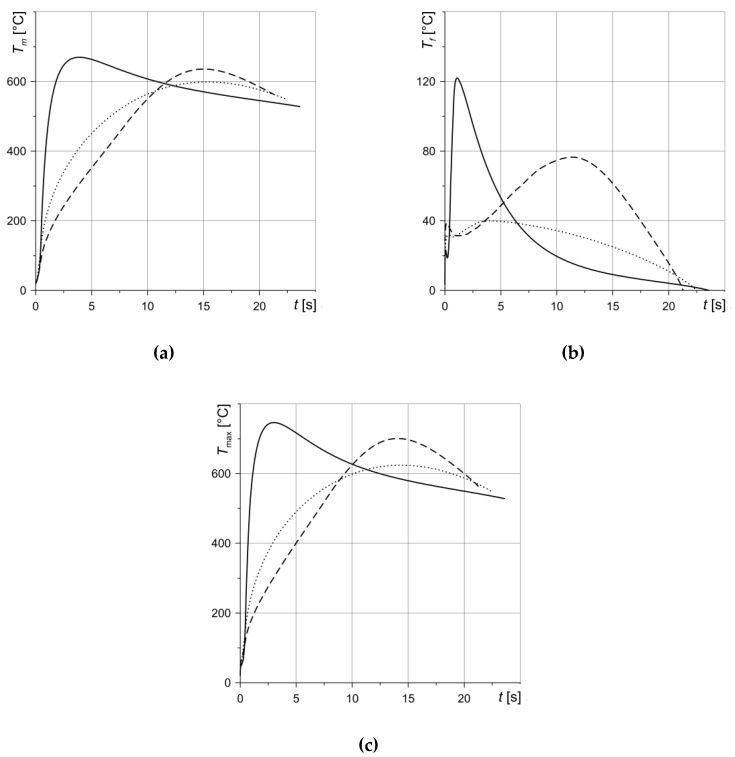
Evolution of the temperature during braking: (**a**) mean *T_m_*; (**b**) flash *T_f_*; (**c**) maximum *T_max_*. Solid line—variant 1, dashed line—variant 2, dotted line—variant 3.

The evolutions of the mean temperature *T_m_* (Equation (24)), flash temperature *T_f_* (Equation (25)) and maximum temperature *T_max_* (Equation (28)) for three calculation variants are presented in Figure 6. We can see that the time course of braking torque *M*, shown in Figure 5b, has a substantial influence on the temperature time profiles. In the case of the first variant, a rapid increase in *M* due to the contact pressure change translates into a corresponding increase in temperature (Figure 6). The monotonic increase in braking torque to reach the maximum value in the middle of the process (second variant) causes similar temperature time profiles. A typical temperature change at the constant deceleration (third option) is characterized by a slight increase at the beginning of the process, reaching a maximum value of about half the braking time and another mild decrease until it stops. The highest temperature *T_max_* values and moments *t_max_* reaching them are as follows: *T_max_* = 746.5 °C, *t_max_* = 3 s (first variant), *T_max_* = 706 °C, *t_max_* = 14.1 s (second variant), *T_max_* = 624 °C, *t_max_* = 14.4 s (third variant). The maximum temperature *T_max_* (Figure 6c) has a decisive influence on the mean temperature *T_m_* (Figure 5a) of the nominal contact area of the rotating and stationary discs. The highest value of the flash temperature *T_f_* = 122 °C is achieved at the time *t_max_* = 1.1 s in the calculations, according to the first variant (Figure 5b).

**Figure 7:**
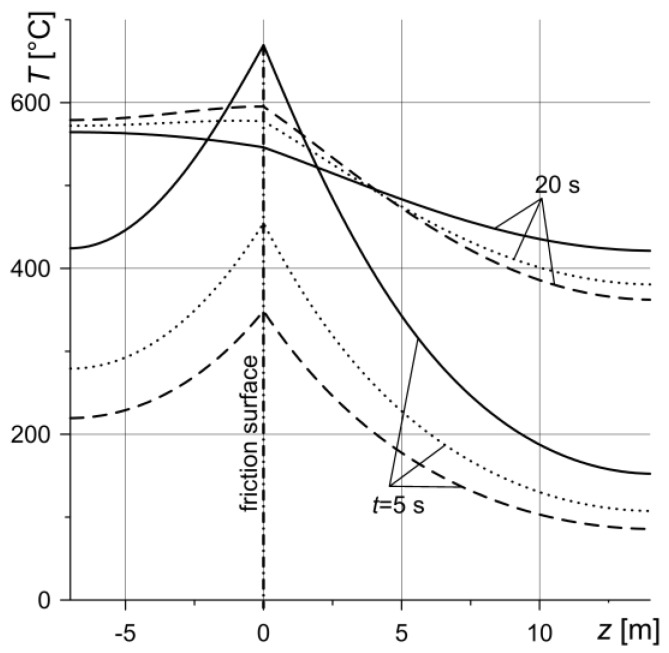
Temperature distributions *T* in axial direction *z* on the equivalent radius *r* = *r_eq_* in two time moments *t* = 5 and 20 s. Solid line—variant 1, dashed line—variant 2, dotted line—variant 3.

The distribution of temperature in the axial direction *z* on the equivalent radius *r* = *r_eq_* at two specified time moments, *t* = 5 and 20 s, during the braking process is shown in Figure 7. According to the adopted assumption of perfect thermal contact of friction (boundary conditions (Equations (14) and (15))), the temperature of the friction surfaces of the discs is equal and shows the highest values. With a distance from the friction surface, the temperature decreases monotonically.

**Figure 8:**
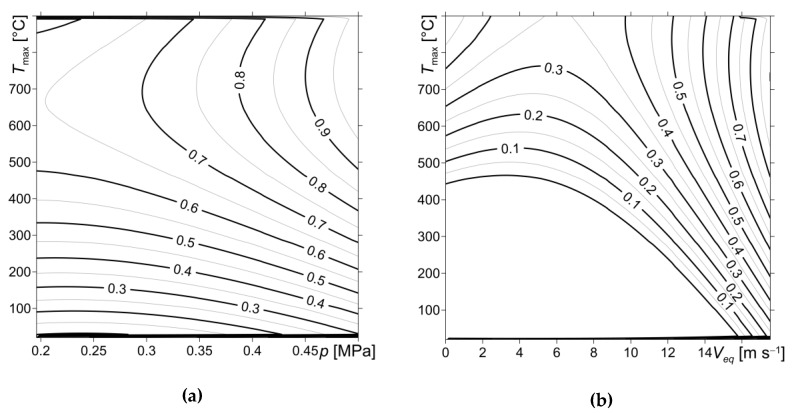
Isolines of the coefficient of friction *f* (variant 1): (**a**) in coordinate system (*p*, *T_max_*) at *V_eq_* = 17.531 m·s^−1^; (**b**) in coordinate system (*V_eq_*, *T_max_*) at *p* = 0.45 MPa.

At a fixed rise time *t_m_* = 0.5 s of contact pressure to the nominal value *p*_0_ = 0.45 MPa, the three-parameter relationship of the coefficient of friction *f* (Equation (29)) allows us to obtain a distribution map of *f* in the orthogonal coordinate system (*p*, *T_max_*) at *V_eq_* = 17.531 m·s^−1^ (Figure 8a). The corresponding map of the coordinates (*V_eq_*, *T_max_*) at *p* = 0.45 MPa is presented in Figure 8b.

## 5. Conclusions

Based on the numerical solution of the HDF system of equations, a methodology for determining the interdependent characteristics of the multi-disc brake has been proposed. The calculations were made for a three-element system, whose discs were made of CFCM-5 material with mechanical and thermophysical properties that change under the influence of temperature. The coefficient of friction was used to combine the initial value problem for the equation of motion and thermal problem of friction contained in the HDF equations. Three variants of that coefficient change based on the experimental data were adapted for numerical calculations in the braking process—three- and single-parameter relationships (variants 1 and 2, respectively) and its constant value, averaged during braking (variant 3). Through our comparative analysis, it was found that the results obtained using the functional dependence of the coefficient of friction on the contact pressure, sliding velocity and maximum temperature (variant 1) are closest to the corresponding experimental data [24]. Another advantage of taking into account the three-parameter dependencies of the coefficient of friction is the ability to use them to obtain maps of the distribution of this parameter at a given velocity (pressure), changing pressure (velocity) and maximum temperature. This method can be a tool for supporting engineers in brake design, particularly in the selection of the optimum temperature mode of operation for a multi-disc system and the selection of the materials used for the discs.

## Figures and Tables

**Table 1:** Approximating coefficients.

K*	*K*_1_	*K*_2_	*K*_3_, °C^−1^	*T_K1_*, °C	*K*_4_	*K*_5_, °C^−1^	*T_K2_*, °C
	−0.7391	0.43056	0.000973932	883.2	1.54951	0.000759595	−252.9
c *	*c*_1_	*c*_2_	*c*_3_, °C^−1^	*T_C1_*, °C	*c*_4_	*c*_5_, °C^−1^	*T_K2_*, °C
	2.6322	0.34628	0.0052908	317.1	−1.7081	0.0032039	9
HB *	*HB*_1_	*HB*_2_	*HB*_3_, °C^−1^	*T_HB1_*, °C	*HB*_4_	*HB*_5_, °C^−1^	*T_HB2_*, °C
	1.623	−8685	35.636	2232	−0.917	0.004	153.762

## Nomenclature

*A_a_*	surface area of the nominal contact region (m^2^)
*A_r_*	total area of the real contact regions (m^2^)
*b*_0_	parameter of the reference-surface curve
*c*	temperature-dependent specific heat capacity (J kg^−1^ K^−1^)
*c*_0_	specific heat capacity at the initial temperature (J kg^−1^ K^−1^)
*d_r_*	diameter of an average spot of real contact (m)
*f*	coefficient of friction
*h*	heat transfer coefficient (W m^−2^ K^−1^)
*h*_max_	maximum roughness height of the friction surface (m)
*HB*	temperature-dependent Brinell hardness (MPa)
*HB*_0_	Brinell hardness at the initial temperature (MPa)
*I*_0_	initial moment of inertia (kg m^2^)
*K*	temperature-dependent thermal conductivity (W m^−1^ K^−1^)
*K*_0_	thermal conductivity at the initial temperature (W m^−1^ K^−1^)
*M*	braking torque (N m)
*p*	contact pressure (MPa)
*p*_0_	nominal contact pressure (MPa)
*q*	friction power density (W m^−2^)
*r*	radial coordinate (m)
*r_av_*	average rounding radius of roughness (m)
*r_eq_*	equivalent radius of the contact region (m)
*t*	time (s)
*t_m_*	rise time (s)
*t_s_*	braking time (s)
*T*	temperature (°C)
*T_a_*	ambient temperature (°C)
*T*_0_	initial temperature (°C)
*T_f_*	flash temperature on the real contact region (°C)
*T_m_*	average temperature of the nominal contact region (°C)
*T_max_*	maximum friction surface temperature (°C)
*V_eq_*	translational velocity on the equivalent radius (m s^−1^)
*W*	work done during braking (J)
*W*_0_	initial rotational kinetic energy (J)
*z*	axial coordinate (m)

Greek symbols

δ	thickness of the single disc of the brake (m)
*v*	parameter of the reference-surface curve
ρ	density (kg m^−3^)
*w*	angular velocity of the rotor (rad s^−1^)
*w*_0_	initial angular velocity of the rotor (rad s^−1^)

Subscripts

*i*	internal
*e*	external
